# [μ-2,8-Dimethyl-1,4,5,6,7,10,11,12-octa­hydro­diimidazo[4,5-*h*;4′,5′-*c*][1,6]diaze­cine-5,11-diacetato]bis­[diaqua­nitrato­copper(II)] trihydrate

**DOI:** 10.1107/S1600536808023969

**Published:** 2008-08-06

**Authors:** Karen S. Luna-Ramírez, Sylvain Bernès, Laura Gasque

**Affiliations:** a Departamento de Química Inorgánica y Nuclear, Facultad de Química, UNAM, 04510 México, DF, Mexico; b DEP Facultad de Ciencias Químicas, UANL, Guerrero y Progreso S/N, Col. Treviño, 64570 Monterrey, NL, Mexico

## Abstract

The title compound, [Cu_2_(C_16_H_20_N_6_O_4_)(NO_3_)_2_(H_2_O)_4_]·3H_2_O, crystallizes with two dinuclear Cu^II^ complex mol­ecules, each lying on an inversion center, and six solvent water mol­ecules per unit cell. The central 1,6-diazecine ring adopts the common chair conformation invariably found in the family of complexes bearing such ligands. The Cu^II^ atoms have an octa­hedral geometry, with a very strong tetra­gonal distortion due to the Jahn–Teller effect. Axial sites are occupied by a nitrate ion and a water mol­ecule. The Cu⋯Cu separations [7.3580 (9) and 7.3341 (9) Å] are compatible with a potential catecholase activity. Neighboring mol­ecules in the crystal structure are connected *via* O—H⋯O hydrogen bonds formed by water mol­ecules and carboxyl­ate O atoms. N—H⋯O hydrogen bonds are also present.

## Related literature

For the X-ray characterized dinuclear Cu^II^ complexes based on related bis­(amino­imidazole) ligands, which were designed as models of the catechol oxidaze active site, see: Driessen *et al.* (2005 ▶); Gasque *et al.* (2005 ▶, 2008 ▶); Mendoza-Díaz *et al.* (2002 ▶); Sosa *et al.* (2005 ▶).
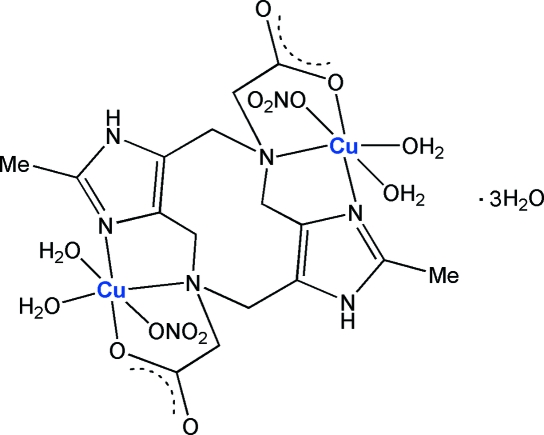


## Experimental

### 

#### Crystal data

[Cu_2_(C_16_H_20_N_6_O_4_)(NO_3_)_2_(H_2_O)_4_]·3H_2_O
*M* *_r_* = 737.59
Triclinic, 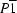

*a* = 7.7983 (9) Å
*b* = 8.7523 (11) Å
*c* = 22.509 (2) Å
α = 91.802 (10)°
β = 93.479 (9)°
γ = 114.023 (11)°
*V* = 1398.0 (3) Å^3^
*Z* = 2
Mo *K*α radiation
μ = 1.61 mm^−1^
*T* = 296 (1) K
0.24 × 0.20 × 0.18 mm

#### Data collection

Bruker P4 diffractometer
Absorption correction: ψ scan (*XSCANS*; Siemens, 1996 ▶) *T*_min_ = 0.638, *T*_max_ = 0.750
12145 measured reflections
6396 independent reflections
5139 reflections with *I* > 2σ(*I*)
*R*_int_ = 0.028
3 standard reflections every 97 reflections intensity decay: 2.5%

#### Refinement

*R*[*F* ^2^ > 2σ(*F* ^2^)] = 0.030
*wR*(*F* ^2^) = 0.082
*S* = 1.02
6396 reflections
433 parameters
22 restraints
H atoms treated by a mixture of independent and constrained refinement
Δρ_max_ = 0.50 e Å^−3^
Δρ_min_ = −0.48 e Å^−3^

### 

Data collection: *XSCANS* (Siemens, 1996 ▶); cell refinement: *XSCANS*; data reduction: *XSCANS*; program(s) used to solve structure: *SHELXS97* (Sheldrick, 2008 ▶); program(s) used to refine structure: *SHELXL97* (Sheldrick, 2008 ▶); molecular graphics: *Mercury* (Macrae *et al.*, 2006 ▶); software used to prepare material for publication: *SHELXL97*.

## Supplementary Material

Crystal structure: contains datablocks I, global. DOI: 10.1107/S1600536808023969/hy2146sup1.cif

Structure factors: contains datablocks I. DOI: 10.1107/S1600536808023969/hy2146Isup2.hkl

Additional supplementary materials:  crystallographic information; 3D view; checkCIF report

## Figures and Tables

**Table 1:** Selected bond lengths (Å)

Cu1—O10	1.9484 (16)
Cu1—O14	1.9533 (16)
Cu1—N1	1.9789 (18)
Cu1—N7	2.0619 (17)
Cu1—O15	2.601 (2)
Cu1—O43	2.607 (2)
Cu2—O30	1.9632 (15)
Cu2—O34	1.9697 (17)
Cu2—N21	1.9903 (18)
Cu2—N27	2.0690 (17)
Cu2—O35	2.4221 (19)
Cu2—O53	2.5994 (19)

**Table 2:** Hydrogen-bond geometry (Å, °)

*D*—H⋯*A*	*D*—H	H⋯*A*	*D*⋯*A*	*D*—H⋯*A*
N3—H3*A*⋯O42^i^	0.86	2.00	2.853 (3)	173
N23—H23*A*⋯O52^ii^	0.86	2.07	2.927 (3)	173
O14—H14*A*⋯O61^iii^	0.84 (1)	1.81 (1)	2.613 (3)	158 (3)
O14—H14*B*⋯O30	0.85 (1)	1.94 (1)	2.782 (2)	178 (3)
O15—H15*A*⋯O31	0.84 (1)	1.89 (1)	2.701 (2)	160 (3)
O15—H15*B*⋯O63^iii^	0.84 (1)	1.98 (2)	2.769 (3)	155 (3)
O34—H34*A*⋯O62	0.84 (1)	1.98 (1)	2.819 (3)	174 (3)
O34—H34*B*⋯O63	0.84 (1)	1.86 (1)	2.665 (3)	160 (3)
O35—H35*A*⋯O10	0.84 (3)	2.02 (3)	2.855 (2)	174 (3)
O35—H35*B*⋯O15^iv^	0.84 (3)	2.11 (3)	2.938 (3)	166 (3)
O61—H61*A*⋯O11^v^	0.84 (3)	1.98 (2)	2.787 (3)	161 (4)
O61—H61*B*⋯O62^iv^	0.85 (3)	2.22 (4)	2.770 (3)	123 (4)
O62—H62*A*⋯O31^v^	0.84 (1)	1.94 (1)	2.777 (3)	173 (4)
O62—H62*B*⋯O52	0.84 (1)	2.24 (2)	3.023 (3)	154 (3)
O63—H63*A*⋯O11^v^	0.84 (1)	1.93 (1)	2.751 (3)	167 (3)
O63—H63*B*⋯O43	0.84 (3)	2.07 (3)	2.898 (3)	170 (3)
O63—H63*B*⋯O42	0.84 (3)	2.49 (2)	3.120 (3)	133 (3)

Symmetry codes: (i) 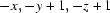; (ii) 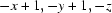; (iii) 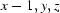; (iv) 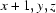; (v) 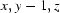.

**Fig. 1.**
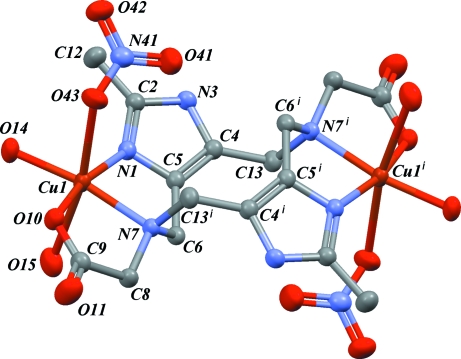
Structure of the first independent molecule. Displacement ellipsoids are drawn at the 50% probability level. H atoms have been omitted for clarity. [Symmetry code: (i) -x, 2-y, 1-z.]

**Fig. 2.**
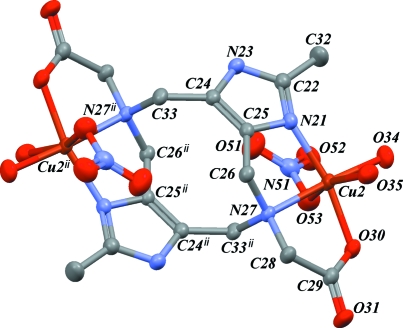
Structure of the second independent molecule. Displacement ellipsoids are drawn at the 50% probability level. H atoms have been omitted for clarity. [Symmetry code: (ii) 1-x, 2-y, -z.]

**Fig. 3.**
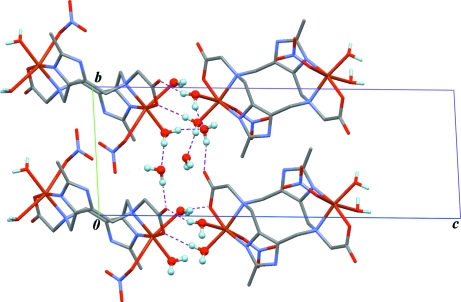
A part of the crystal packing, viewed down the [100] direction. C- and N–bonded H atoms have been omitted for clarity. Dashed lines represent hydrogen bonds involving water molecules and carboxylate O atoms.

## supplementary crystallographic information

### Comment

Several dinucleating ligands containing imidazole and amines condensed *via* the Mannich reaction have been recently described, as well as their dicopper complexes (Driessen *et al.*, 2005; Gasque *et al.*, 2005; Mendoza-Díaz *et al.*, 2002; Sosa *et al.*, 2005), which exhibit interesting magnetic properties and a significant catecholase activity (Gasque *et al.*, 2008). The complex presented here is an analogue of the first dicopper complex of this family reported (Mendoza-Díaz *et al.*, 2002), in which perchlorate ion has been substituted by nitrate. As nitrate ions have a stronger ability to coordinate metal ions, the present complex is found to have six-coordinated Cu^II^ centers, while perchlorate ions afforded a cationic complex including five-coordinated metal centers.

The asymmetric unit of the title compound is formed by two half-complexes, placed close to inversion centers, and three lattice water molecules, lying on general positions. The triclinic unit cell thus contains two dinuclar centrosymmetric complexes (Figs. 1 and 2), which have similar structures. The bis(aminoimidazole) ligand coordinates two Cu^II^ atoms, *via* imidazole and tertiary N atoms, and one carboxylate O atom. Each Cu^II^ atom completes its coordination environment with two water molecules and one monodentate nitrate ion. The resulting coordination geometry is octahedral, with a very strong distortion due to the Jahn-Teller effect: Cu—O axial bond lengths are in the range of 2.4221 (19)–2.607 (2) Å, while the longest equatorial bond measures 2.0690 (17) Å (Table 1). The central 1,6-diazecine 10-membered ring displays a chair conformation, with a total puckering amplitude of 1.441 (2) and 1.434 (2) Å for Cu1- and Cu2-complex, respectively. The Cu···Cu separations, 7.3580 (9) and 7.3341 (9) Å, are probably compatible with a catecholase activity for this molecular compound (Gasque *et al.*, 2008).

Lattice water molecules are active into forming a number of rather strong hydrogen bonds (Table 2), connecting neighboring molecules in the crystal. Strongest hydrogen bonds involve all water molecules, as both donor and acceptor, and carboxylate O atoms as acceptor groups (Fig. 3).

### Experimental

The diazecine derivative was prepared as described previously (Mendoza-Díaz *et al.*, 2002). To prepare the title compound, Cu(NO_3_)_2_.2.5H_2_O (0.232 g, 1 mmol) were dissolved in 20 ml of water, and an aqueous solution containing the ligand (0.251 g, 0.5 mmol) was added dropwise with stirring. The final pH was 2, at which the solution was left to stand. Blue crystals were collected after two days. Analysis, calculated for C_16_H_34_Cu_2_N_8_O_17_: C 26.05, H 4.61, N 15.20%; found: C 26.67, H 4.55, N 15.55%.

### Refinement

Water H atoms were located in a difference Fourier map and refined with distance restraints of O—H = 0.85 (1) and H···H = 1.34 (1) Å, and with *U*_iso_(H) = 1.5*U*_eq_(O). Other H atoms were positioned geometrically and refined as riding atoms, with N—H = 0.86 and C—H = 0.96 (CH_3_) and 0.97 Å (CH_2_), and with *U*_iso_(H) = 1.2*U*_eq_(C,N) or *U*_iso_(H) = 1.5*U*_eq_(methyl C).

### Crystal data

**Table:**

[Cu_2_(C_16_H_20_N_6_O_4_)(NO_3_)_2_(H_2_O)_4_]·3H_2_O	*Z* = 2
*M**_r_* = 737.59	*F*_000_ = 760
Triclinic, *P*1	*D*_x_ = 1.752 Mg m^−^^3^
Hall symbol: -P 1	Mo *K*α radiation λ = 0.71073 Å
*a* = 7.7983 (9) Å	Cell parameters from 74 reflections
*b* = 8.7523 (11) Å	θ = 3.8–12.5º
*c* = 22.509 (2) Å	µ = 1.61 mm^−^^1^
α = 91.802 (10)º	*T* = 296 (1) K
β = 93.479 (9)º	Prism, blue
γ = 114.023 (11)º	0.24 × 0.20 × 0.18 mm
*V* = 1398.0 (3) Å^3^	

### Data collection

**Table:**

Bruker P4 diffractometer	*R*_int_ = 0.028
Radiation source: fine-focus sealed tube	θ_max_ = 27.5º
Monochromator: graphite	θ_min_ = 2.6º
*T* = 296(1) K	*h* = −9→7
2θ/ω scans	*k* = −10→11
Absorption correction: ψ scan(XSCANS; Siemens, 1996)	*l* = −29→29
*T*_min_ = 0.638, *T*_max_ = 0.750	3 standard reflections
12145 measured reflections	every 97 reflections
6396 independent reflections	intensity decay: 2.5%
5139 reflections with *I* > 2σ(*I*)	

### Refinement

**Table:**

Refinement on *F*^2^	Secondary atom site location: difference Fourier map
Least-squares matrix: full	Hydrogen site location: inferred from neighbouring sites
*R*[*F*^2^ > 2σ(*F*^2^)] = 0.031	H atoms treated by a mixture of independent and constrained refinement
*wR*(*F*^2^) = 0.082	*w* = 1/[σ^2^(*F*_o_^2^) + (0.0357*P*)^2^ + 0.6951*P*] where *P* = (*F*_o_^2^ + 2*F*_c_^2^)/3
*S* = 1.02	(Δ/σ)_max_ = 0.002
6396 reflections	Δρ_max_ = 0.50 e Å^−^^3^
433 parameters	Δρ_min_ = −0.48 e Å^−^^3^
22 restraints	Extinction correction: SHELXL97 (Sheldrick, 2008), Fc^*^=kFc[1+0.001xFc^2^λ^3^/sin(2θ)]^-1/4^
Primary atom site location: structure-invariant direct methods	Extinction coefficient: 0.0028 (5)

### Fractional atomic coordinates and isotropic or equivalent isotropic displacement parameters (Å^2^)

**Table:**

	*x*	*y*	*z*	*U*_iso_*/*U*_eq_	
Cu1	0.08644 (4)	0.89792 (3)	0.348009 (11)	0.02579 (9)	
N1	−0.0982 (3)	0.7671 (2)	0.40361 (8)	0.0248 (4)	
C2	−0.1523 (3)	0.6214 (3)	0.42879 (10)	0.0256 (4)	
N3	−0.2240 (3)	0.6329 (2)	0.48134 (8)	0.0258 (4)	
H3A	−0.2680	0.5535	0.5052	0.031*	
C4	−0.2147 (3)	0.7941 (3)	0.49036 (9)	0.0238 (4)	
C5	−0.1392 (3)	0.8745 (3)	0.44130 (9)	0.0231 (4)	
C6	−0.0845 (3)	1.0489 (3)	0.42218 (9)	0.0234 (4)	
H6A	−0.0850	1.1228	0.4551	0.028*	
H6B	−0.1710	1.0513	0.3898	0.028*	
N7	0.1104 (3)	1.1013 (2)	0.40212 (8)	0.0236 (4)	
C8	0.1656 (3)	1.2389 (3)	0.36114 (10)	0.0275 (5)	
H8A	0.2246	1.3460	0.3838	0.033*	
H8B	0.0550	1.2358	0.3380	0.033*	
C9	0.3037 (3)	1.2185 (3)	0.31942 (10)	0.0294 (5)	
O10	0.2858 (2)	1.06789 (19)	0.30833 (7)	0.0303 (3)	
O11	0.4199 (3)	1.3415 (2)	0.29801 (9)	0.0502 (5)	
C12	−0.1377 (4)	0.4685 (3)	0.40444 (12)	0.0388 (6)	
H12A	−0.0285	0.4996	0.3822	0.058*	
H12B	−0.2484	0.4038	0.3787	0.058*	
H12C	−0.1267	0.4028	0.4366	0.058*	
C13	−0.2580 (3)	0.8638 (3)	0.54610 (9)	0.0262 (4)	
H13A	−0.3785	0.7852	0.5578	0.031*	
H13B	−0.2700	0.9673	0.5377	0.031*	
O14	0.0602 (3)	0.7270 (2)	0.28594 (7)	0.0337 (4)	
H14A	−0.048 (2)	0.651 (3)	0.2794 (12)	0.051*	
H14B	0.087 (4)	0.770 (3)	0.2528 (7)	0.051*	
O15	−0.1735 (3)	0.9468 (2)	0.28392 (8)	0.0421 (4)	
H15A	−0.126 (4)	0.955 (4)	0.2509 (8)	0.063*	
H15B	−0.263 (3)	0.851 (2)	0.2816 (14)	0.063*	
Cu2	0.35494 (4)	0.83718 (3)	0.145370 (11)	0.02455 (8)	
N21	0.5757 (3)	0.8507 (2)	0.10232 (8)	0.0246 (4)	
C22	0.6423 (3)	0.7446 (3)	0.08044 (10)	0.0273 (5)	
N23	0.7269 (3)	0.8023 (2)	0.03001 (8)	0.0258 (4)	
H23A	0.7806	0.7542	0.0086	0.031*	
C24	0.7128 (3)	0.9526 (3)	0.01846 (9)	0.0225 (4)	
C25	0.6218 (3)	0.9813 (3)	0.06424 (9)	0.0218 (4)	
C26	0.5580 (3)	1.1153 (3)	0.08005 (9)	0.0225 (4)	
H26A	0.5708	1.1879	0.0474	0.027*	
H26B	0.6314	1.1827	0.1152	0.027*	
N27	0.3561 (2)	1.0257 (2)	0.09200 (7)	0.0213 (3)	
C28	0.2817 (3)	1.1241 (3)	0.12900 (10)	0.0267 (5)	
H28A	0.2282	1.1836	0.1036	0.032*	
H28B	0.3831	1.2059	0.1552	0.032*	
C29	0.1307 (3)	1.0060 (3)	0.16603 (10)	0.0278 (5)	
O30	0.1386 (2)	0.86562 (19)	0.17597 (7)	0.0293 (3)	
O31	0.0133 (3)	1.0514 (2)	0.18558 (8)	0.0415 (4)	
C32	0.6350 (4)	0.5899 (3)	0.10759 (12)	0.0416 (6)	
H32A	0.5864	0.5831	0.1460	0.062*	
H32B	0.7595	0.5927	0.1119	0.062*	
H32C	0.5544	0.4937	0.0823	0.062*	
C33	0.7703 (3)	1.0501 (3)	−0.03551 (9)	0.0248 (4)	
H33A	0.8968	1.0630	−0.0427	0.030*	
H33B	0.7751	1.1612	−0.0272	0.030*	
O34	0.3272 (3)	0.6462 (2)	0.19384 (8)	0.0351 (4)	
H34A	0.222 (2)	0.564 (3)	0.1904 (12)	0.053*	
H34B	0.364 (4)	0.668 (4)	0.2303 (6)	0.053*	
O35	0.5368 (3)	1.0386 (2)	0.22603 (8)	0.0393 (4)	
H35A	0.466 (3)	1.044 (4)	0.2520 (10)	0.059*	
H35B	0.628 (3)	1.029 (4)	0.2450 (12)	0.059*	
N41	0.3385 (3)	0.7364 (2)	0.43818 (9)	0.0332 (4)	
O41	0.2854 (4)	0.7699 (3)	0.48506 (9)	0.0635 (6)	
O42	0.3741 (3)	0.6098 (2)	0.43267 (9)	0.0426 (4)	
O43	0.3583 (3)	0.8284 (3)	0.39489 (8)	0.0487 (5)	
N51	0.1540 (3)	0.4932 (2)	0.04767 (9)	0.0320 (4)	
O51	0.2721 (3)	0.5612 (3)	0.01204 (9)	0.0540 (5)	
O52	0.0845 (3)	0.3372 (2)	0.05034 (9)	0.0449 (5)	
O53	0.1045 (3)	0.5808 (2)	0.08235 (8)	0.0425 (4)	
O61	0.7678 (4)	0.4526 (3)	0.24970 (14)	0.0846 (10)	
H61A	0.657 (3)	0.398 (5)	0.259 (2)	0.127*	
H61B	0.819 (4)	0.384 (3)	0.250 (2)	0.127*	
O62	−0.0134 (3)	0.3570 (2)	0.17744 (9)	0.0484 (5)	
H62A	−0.015 (5)	0.260 (2)	0.1804 (14)	0.073*	
H62B	−0.006 (5)	0.371 (4)	0.1406 (6)	0.073*	
O63	0.4948 (3)	0.6773 (2)	0.30304 (8)	0.0404 (4)	
H63A	0.477 (5)	0.5775 (18)	0.3072 (14)	0.061*	
H63B	0.448 (5)	0.709 (4)	0.3310 (11)	0.061*	

### Atomic displacement parameters (Å^2^)

**Table:**

	*U*^11^	*U*^22^	*U*^33^	*U*^12^	*U*^13^	*U*^23^
Cu1	0.03386 (16)	0.02060 (13)	0.02290 (14)	0.00971 (11)	0.01201 (11)	0.00325 (10)
N1	0.0294 (10)	0.0231 (9)	0.0225 (9)	0.0105 (8)	0.0069 (7)	0.0032 (7)
C2	0.0292 (11)	0.0233 (10)	0.0246 (11)	0.0104 (9)	0.0057 (9)	0.0047 (8)
N3	0.0300 (10)	0.0217 (9)	0.0246 (9)	0.0082 (7)	0.0082 (7)	0.0066 (7)
C4	0.0232 (10)	0.0216 (10)	0.0250 (10)	0.0069 (8)	0.0054 (8)	0.0026 (8)
C5	0.0225 (10)	0.0228 (10)	0.0233 (10)	0.0081 (8)	0.0043 (8)	0.0021 (8)
C6	0.0256 (11)	0.0249 (10)	0.0220 (10)	0.0119 (9)	0.0060 (8)	0.0038 (8)
N7	0.0294 (10)	0.0211 (8)	0.0214 (9)	0.0103 (7)	0.0089 (7)	0.0046 (7)
C8	0.0361 (12)	0.0221 (10)	0.0256 (11)	0.0117 (9)	0.0111 (9)	0.0064 (8)
C9	0.0338 (12)	0.0280 (11)	0.0265 (11)	0.0114 (10)	0.0102 (9)	0.0064 (9)
O10	0.0363 (9)	0.0257 (8)	0.0298 (8)	0.0116 (7)	0.0150 (7)	0.0051 (6)
O11	0.0573 (12)	0.0308 (9)	0.0619 (13)	0.0124 (9)	0.0368 (10)	0.0140 (9)
C12	0.0554 (16)	0.0271 (12)	0.0382 (14)	0.0197 (11)	0.0143 (12)	0.0054 (10)
C13	0.0254 (11)	0.0285 (11)	0.0248 (11)	0.0101 (9)	0.0085 (8)	0.0034 (8)
O14	0.0437 (10)	0.0272 (8)	0.0259 (8)	0.0088 (7)	0.0127 (7)	0.0000 (7)
O15	0.0395 (11)	0.0469 (11)	0.0327 (9)	0.0092 (8)	0.0114 (8)	0.0047 (8)
Cu2	0.02862 (15)	0.02653 (14)	0.02214 (14)	0.01350 (11)	0.00965 (10)	0.00828 (10)
N21	0.0261 (9)	0.0271 (9)	0.0228 (9)	0.0123 (8)	0.0054 (7)	0.0070 (7)
C22	0.0281 (11)	0.0315 (11)	0.0257 (11)	0.0148 (9)	0.0056 (9)	0.0064 (9)
N23	0.0288 (10)	0.0296 (9)	0.0242 (9)	0.0163 (8)	0.0076 (7)	0.0040 (7)
C24	0.0211 (10)	0.0252 (10)	0.0218 (10)	0.0098 (8)	0.0032 (8)	0.0037 (8)
C25	0.0205 (10)	0.0237 (10)	0.0209 (10)	0.0086 (8)	0.0026 (8)	0.0044 (8)
C26	0.0230 (10)	0.0224 (10)	0.0220 (10)	0.0085 (8)	0.0055 (8)	0.0035 (8)
N27	0.0230 (9)	0.0220 (8)	0.0201 (8)	0.0097 (7)	0.0067 (7)	0.0032 (7)
C28	0.0314 (12)	0.0297 (11)	0.0243 (10)	0.0166 (9)	0.0099 (9)	0.0052 (9)
C29	0.0300 (12)	0.0333 (12)	0.0226 (11)	0.0145 (10)	0.0075 (9)	0.0046 (9)
O30	0.0343 (9)	0.0302 (8)	0.0265 (8)	0.0146 (7)	0.0128 (6)	0.0076 (6)
O31	0.0460 (11)	0.0491 (11)	0.0446 (10)	0.0310 (9)	0.0265 (8)	0.0172 (8)
C32	0.0576 (17)	0.0428 (14)	0.0415 (14)	0.0350 (13)	0.0172 (12)	0.0187 (12)
C33	0.0215 (10)	0.0290 (11)	0.0228 (10)	0.0083 (9)	0.0061 (8)	0.0053 (8)
O34	0.0404 (10)	0.0308 (9)	0.0314 (9)	0.0109 (7)	0.0053 (7)	0.0111 (7)
O35	0.0399 (10)	0.0510 (11)	0.0328 (9)	0.0241 (9)	0.0069 (8)	0.0005 (8)
N41	0.0346 (11)	0.0308 (10)	0.0348 (11)	0.0136 (9)	0.0039 (9)	0.0068 (8)
O41	0.1023 (19)	0.0726 (15)	0.0410 (12)	0.0576 (14)	0.0303 (12)	0.0161 (10)
O42	0.0498 (11)	0.0284 (9)	0.0530 (11)	0.0177 (8)	0.0129 (9)	0.0093 (8)
O43	0.0711 (14)	0.0503 (11)	0.0392 (10)	0.0368 (11)	0.0161 (9)	0.0207 (9)
N51	0.0358 (11)	0.0319 (10)	0.0311 (10)	0.0174 (9)	−0.0023 (8)	−0.0005 (8)
O51	0.0574 (13)	0.0541 (12)	0.0461 (12)	0.0165 (10)	0.0171 (10)	0.0033 (9)
O52	0.0558 (12)	0.0292 (9)	0.0502 (11)	0.0183 (8)	0.0008 (9)	0.0021 (8)
O53	0.0532 (12)	0.0388 (10)	0.0416 (10)	0.0249 (9)	0.0074 (9)	−0.0008 (8)
O61	0.0627 (16)	0.0583 (15)	0.101 (2)	−0.0102 (12)	0.0429 (15)	−0.0296 (15)
O62	0.0574 (12)	0.0403 (11)	0.0497 (12)	0.0196 (10)	0.0189 (10)	0.0102 (9)
O63	0.0469 (11)	0.0366 (10)	0.0365 (10)	0.0144 (9)	0.0103 (8)	0.0094 (8)

### Geometric parameters (Å, °)

**Table:**

Cu1—O10	1.9484 (16)	C33—N27^ii^	1.514 (3)
Cu1—O14	1.9533 (16)	N41—O41	1.226 (3)
Cu1—N1	1.9789 (18)	N41—O42	1.251 (3)
Cu1—N7	2.0619 (17)	N41—O43	1.260 (3)
Cu1—O15	2.601 (2)	N51—O51	1.233 (3)
Cu1—O43	2.607 (2)	N51—O52	1.253 (3)
N1—C2	1.327 (3)	N51—O53	1.259 (3)
N1—C5	1.390 (3)	N3—H3A	0.8600
C2—N3	1.357 (3)	C6—H6A	0.9700
C2—C12	1.480 (3)	C6—H6B	0.9700
N3—C4	1.391 (3)	C8—H8A	0.9700
C4—C5	1.361 (3)	C8—H8B	0.9700
C4—C13	1.494 (3)	C12—H12A	0.9600
C5—C6	1.493 (3)	C12—H12B	0.9600
C6—N7	1.501 (3)	C12—H12C	0.9600
N7—C8	1.477 (3)	C13—H13A	0.9700
N7—C13^i^	1.519 (3)	C13—H13B	0.9700
C8—C9	1.530 (3)	O14—H14A	0.84 (1)
C9—O11	1.226 (3)	O14—H14B	0.85 (1)
C9—O10	1.282 (3)	O15—H15A	0.84 (1)
C13—N7^i^	1.519 (3)	O15—H15B	0.84 (1)
Cu2—O30	1.9632 (15)	N23—H23A	0.8600
Cu2—O34	1.9697 (17)	C26—H26A	0.9700
Cu2—N21	1.9903 (18)	C26—H26B	0.9700
Cu2—N27	2.0690 (17)	C28—H28A	0.9700
Cu2—O35	2.4221 (19)	C28—H28B	0.9700
Cu2—O53	2.5994 (19)	C32—H32A	0.9600
N21—C22	1.330 (3)	C32—H32B	0.9600
N21—C25	1.391 (3)	C32—H32C	0.9600
C22—N23	1.355 (3)	C33—H33A	0.9700
C22—C32	1.484 (3)	C33—H33B	0.9700
N23—C24	1.395 (3)	O34—H34A	0.84 (1)
C24—C25	1.359 (3)	O34—H34B	0.84 (1)
C24—C33	1.492 (3)	O35—H35A	0.84 (3)
C25—C26	1.490 (3)	O35—H35B	0.84 (3)
C26—N27	1.492 (3)	O61—H61A	0.84 (3)
N27—C28	1.480 (3)	O61—H61B	0.85 (3)
N27—C33^ii^	1.514 (3)	O62—H62A	0.84 (1)
C28—C29	1.527 (3)	O62—H62B	0.84 (1)
C29—O31	1.234 (3)	O63—H63A	0.84 (1)
C29—O30	1.281 (3)	O63—H63B	0.84 (3)
			
O10—Cu1—O14	91.21 (7)	N27—C28—C29	109.37 (17)
O10—Cu1—N1	166.48 (7)	O31—C29—O30	124.2 (2)
O14—Cu1—N1	102.13 (7)	O31—C29—C28	119.3 (2)
O10—Cu1—N7	82.87 (7)	O30—C29—C28	116.51 (19)
O14—Cu1—N7	170.59 (7)	C29—O30—Cu2	114.94 (14)
N1—Cu1—N7	84.17 (7)	C24—C33—N27^ii^	115.46 (17)
O10—Cu1—O15	91.86 (7)	O41—N41—O42	120.3 (2)
O14—Cu1—O15	86.02 (7)	O41—N41—O43	120.8 (2)
N1—Cu1—O15	91.24 (7)	O42—N41—O43	118.9 (2)
N7—Cu1—O15	86.87 (7)	N41—O43—Cu1	122.73 (16)
O10—Cu1—O43	84.37 (7)	O51—N51—O52	120.4 (2)
O14—Cu1—O43	84.84 (7)	O51—N51—O53	120.0 (2)
N1—Cu1—O43	94.57 (7)	O52—N51—O53	119.5 (2)
N7—Cu1—O43	101.77 (7)	N51—O53—Cu2	120.63 (15)
O15—Cu1—O43	170.03 (6)	C2—N3—H3A	125.6
C2—N1—C5	106.92 (17)	C4—N3—H3A	125.6
C2—N1—Cu1	139.08 (15)	C5—C6—H6A	110.6
C5—N1—Cu1	109.98 (13)	N7—C6—H6A	110.6
N1—C2—N3	109.50 (18)	C5—C6—H6B	110.6
N1—C2—C12	126.2 (2)	N7—C6—H6B	110.6
N3—C2—C12	124.3 (2)	H6A—C6—H6B	108.8
C2—N3—C4	108.79 (17)	N7—C8—H8A	109.9
C5—C4—N3	105.16 (18)	C9—C8—H8A	109.9
C5—C4—C13	128.95 (19)	N7—C8—H8B	109.9
N3—C4—C13	125.51 (19)	C9—C8—H8B	109.9
C4—C5—N1	109.61 (18)	H8A—C8—H8B	108.3
C4—C5—C6	134.29 (19)	C2—C12—H12A	109.5
N1—C5—C6	116.00 (18)	C2—C12—H12B	109.5
C5—C6—N7	105.46 (16)	H12A—C12—H12B	109.5
C8—N7—C6	114.66 (17)	C2—C12—H12C	109.5
C8—N7—C13^i^	111.73 (17)	H12A—C12—H12C	109.5
C6—N7—C13^i^	112.48 (16)	H12B—C12—H12C	109.5
C8—N7—Cu1	102.92 (12)	C4—C13—H13A	108.8
C6—N7—Cu1	102.80 (12)	N7^i^—C13—H13A	108.8
C13^i^—N7—Cu1	111.46 (13)	C4—C13—H13B	108.8
N7—C8—C9	108.93 (17)	N7^i^—C13—H13B	108.8
O11—C9—O10	124.0 (2)	H13A—C13—H13B	107.7
O11—C9—C8	120.1 (2)	Cu1—O14—H14A	115.2 (19)
O10—C9—C8	115.84 (19)	Cu1—O14—H14B	111 (2)
C9—O10—Cu1	114.73 (14)	H14A—O14—H14B	105 (2)
C4—C13—N7^i^	113.84 (18)	Cu1—O15—H15A	98 (2)
O30—Cu2—O34	94.11 (7)	Cu1—O15—H15B	102 (2)
O30—Cu2—N21	166.65 (7)	H15A—O15—H15B	105 (2)
O34—Cu2—N21	99.21 (7)	C22—N23—H23A	125.6
O30—Cu2—N27	82.21 (7)	C24—N23—H23A	125.6
O34—Cu2—N27	174.38 (7)	C25—C26—H26A	110.6
N21—Cu2—N27	84.44 (7)	N27—C26—H26A	110.6
O30—Cu2—O35	84.72 (7)	C25—C26—H26B	110.6
O34—Cu2—O35	92.25 (7)	N27—C26—H26B	110.6
N21—Cu2—O35	95.62 (7)	H26A—C26—H26B	108.8
N27—Cu2—O35	91.64 (7)	N27—C28—H28A	109.8
O30—Cu2—O53	85.36 (6)	C29—C28—H28A	109.8
O34—Cu2—O53	76.31 (7)	N27—C28—H28B	109.8
N21—Cu2—O53	96.84 (7)	C29—C28—H28B	109.8
N27—Cu2—O53	99.08 (6)	H28A—C28—H28B	108.2
O35—Cu2—O53	164.27 (6)	C22—C32—H32A	109.5
C22—N21—C25	106.61 (17)	C22—C32—H32B	109.5
C22—N21—Cu2	137.21 (15)	H32A—C32—H32B	109.5
C25—N21—Cu2	109.28 (13)	C22—C32—H32C	109.5
N21—C22—N23	109.61 (19)	H32A—C32—H32C	109.5
N21—C22—C32	126.3 (2)	H32B—C32—H32C	109.5
N23—C22—C32	124.1 (2)	C24—C33—H33A	108.4
C22—N23—C24	108.84 (18)	N27^ii^—C33—H33A	108.4
C25—C24—N23	104.89 (18)	C24—C33—H33B	108.4
C25—C24—C33	128.23 (19)	N27^ii^—C33—H33B	108.4
N23—C24—C33	126.66 (19)	H33A—C33—H33B	107.5
C24—C25—N21	110.02 (18)	Cu2—O34—H34A	117 (2)
C24—C25—C26	134.12 (19)	Cu2—O34—H34B	117 (2)
N21—C25—C26	115.81 (17)	H34A—O34—H34B	109 (2)
C25—C26—N27	105.47 (16)	Cu2—O35—H35A	110 (2)
C28—N27—C26	114.94 (16)	Cu2—O35—H35B	121 (2)
C28—N27—C33^ii^	111.27 (17)	H35A—O35—H35B	105 (2)
C26—N27—C33^ii^	112.37 (15)	H61A—O61—H61B	106 (2)
C28—N27—Cu2	104.14 (12)	H62A—O62—H62B	103 (2)
C26—N27—Cu2	103.68 (12)	H63A—O63—H63B	108 (2)
C33^ii^—N27—Cu2	109.74 (12)		
			
O10—Cu1—N1—C2	−126.5 (3)	N27—Cu2—N21—C25	4.30 (14)
O14—Cu1—N1—C2	44.1 (2)	O35—Cu2—N21—C25	−86.81 (14)
N7—Cu1—N1—C2	−143.0 (2)	O53—Cu2—N21—C25	102.81 (14)
O15—Cu1—N1—C2	130.3 (2)	C25—N21—C22—N23	−0.5 (2)
O43—Cu1—N1—C2	−41.6 (2)	Cu2—N21—C22—N23	145.56 (18)
O10—Cu1—N1—C5	26.7 (4)	C25—N21—C22—C32	176.9 (2)
O14—Cu1—N1—C5	−162.71 (14)	Cu2—N21—C22—C32	−37.1 (4)
N7—Cu1—N1—C5	10.21 (14)	N21—C22—N23—C24	−0.5 (3)
O15—Cu1—N1—C5	−76.52 (14)	C32—C22—N23—C24	−178.0 (2)
O43—Cu1—N1—C5	111.60 (14)	C22—N23—C24—C25	1.3 (2)
C5—N1—C2—N3	−0.7 (2)	C22—N23—C24—C33	−173.7 (2)
Cu1—N1—C2—N3	152.96 (18)	N23—C24—C25—N21	−1.6 (2)
C5—N1—C2—C12	179.5 (2)	C33—C24—C25—N21	173.3 (2)
Cu1—N1—C2—C12	−26.8 (4)	N23—C24—C25—C26	−179.1 (2)
N1—C2—N3—C4	−0.2 (3)	C33—C24—C25—C26	−4.2 (4)
C12—C2—N3—C4	179.6 (2)	C22—N21—C25—C24	1.3 (2)
C2—N3—C4—C5	1.1 (2)	Cu2—N21—C25—C24	−154.94 (15)
C2—N3—C4—C13	−172.3 (2)	C22—N21—C25—C26	179.31 (18)
N3—C4—C5—N1	−1.5 (2)	Cu2—N21—C25—C26	23.0 (2)
C13—C4—C5—N1	171.5 (2)	C24—C25—C26—N27	129.3 (2)
N3—C4—C5—C6	−177.7 (2)	N21—C25—C26—N27	−48.0 (2)
C13—C4—C5—C6	−4.7 (4)	C25—C26—N27—C28	158.64 (17)
C2—N1—C5—C4	1.4 (2)	C25—C26—N27—C33^ii^	−72.8 (2)
Cu1—N1—C5—C4	−160.57 (15)	C25—C26—N27—Cu2	45.67 (16)
C2—N1—C5—C6	178.42 (19)	O30—Cu2—N27—C28	30.55 (13)
Cu1—N1—C5—C6	16.4 (2)	N21—Cu2—N27—C28	−149.39 (14)
C4—C5—C6—N7	131.6 (3)	O35—Cu2—N27—C28	−53.90 (13)
N1—C5—C6—N7	−44.5 (2)	O53—Cu2—N27—C28	114.55 (13)
C5—C6—N7—C8	158.05 (17)	O30—Cu2—N27—C26	151.13 (12)
C5—C6—N7—C13^i^	−72.8 (2)	N21—Cu2—N27—C26	−28.80 (12)
C5—C6—N7—Cu1	47.16 (17)	O35—Cu2—N27—C26	66.69 (12)
O10—Cu1—N7—C8	31.67 (14)	O53—Cu2—N27—C26	−124.87 (12)
N1—Cu1—N7—C8	−152.16 (14)	O30—Cu2—N27—C33^ii^	−88.64 (13)
O15—Cu1—N7—C8	−60.59 (13)	N21—Cu2—N27—C33^ii^	91.42 (13)
O43—Cu1—N7—C8	114.37 (13)	O35—Cu2—N27—C33^ii^	−173.09 (12)
O10—Cu1—N7—C6	151.08 (13)	O53—Cu2—N27—C33^ii^	−4.64 (13)
N1—Cu1—N7—C6	−32.75 (13)	C26—N27—C28—C29	−148.41 (18)
O15—Cu1—N7—C6	58.82 (12)	C33^ii^—N27—C28—C29	82.4 (2)
O43—Cu1—N7—C6	−126.22 (12)	Cu2—N27—C28—C29	−35.70 (19)
O10—Cu1—N7—C13^i^	−88.21 (13)	N27—C28—C29—O31	−158.3 (2)
N1—Cu1—N7—C13^i^	87.95 (13)	N27—C28—C29—O30	23.4 (3)
O15—Cu1—N7—C13^i^	179.52 (13)	O31—C29—O30—Cu2	−174.47 (19)
O43—Cu1—N7—C13^i^	−5.51 (14)	C28—C29—O30—Cu2	3.7 (2)
C6—N7—C8—C9	−149.67 (18)	O34—Cu2—O30—C29	164.11 (16)
C13^i^—N7—C8—C9	80.8 (2)	N21—Cu2—O30—C29	−19.9 (4)
Cu1—N7—C8—C9	−38.8 (2)	N27—Cu2—O30—C29	−20.16 (15)
N7—C8—C9—O11	−152.6 (2)	O35—Cu2—O30—C29	72.23 (16)
N7—C8—C9—O10	28.6 (3)	O53—Cu2—O30—C29	−120.00 (16)
O11—C9—O10—Cu1	−179.2 (2)	C25—C24—C33—N27^ii^	−101.2 (3)
C8—C9—O10—Cu1	−0.5 (3)	N23—C24—C33—N27^ii^	72.7 (3)
O14—Cu1—O10—C9	154.16 (17)	O41—N41—O43—Cu1	−56.3 (3)
N1—Cu1—O10—C9	−35.0 (4)	O42—N41—O43—Cu1	123.81 (19)
N7—Cu1—O10—C9	−18.51 (16)	O10—Cu1—O43—N41	167.17 (19)
O15—Cu1—O10—C9	68.10 (17)	O14—Cu1—O43—N41	−101.09 (19)
O43—Cu1—O10—C9	−121.15 (17)	N1—Cu1—O43—N41	0.70 (19)
C5—C4—C13—N7^i^	−99.9 (3)	N7—Cu1—O43—N41	85.68 (19)
N3—C4—C13—N7^i^	71.9 (3)	O51—N51—O53—Cu2	−49.7 (3)
O30—Cu2—N21—C22	−141.4 (3)	O52—N51—O53—Cu2	129.25 (19)
O34—Cu2—N21—C22	34.5 (2)	O30—Cu2—O53—N51	179.93 (17)
N27—Cu2—N21—C22	−141.1 (2)	O34—Cu2—O53—N51	−84.69 (17)
O35—Cu2—N21—C22	127.8 (2)	N21—Cu2—O53—N51	13.17 (18)
O53—Cu2—N21—C22	−42.6 (2)	N27—Cu2—O53—N51	98.60 (17)
O30—Cu2—N21—C25	4.0 (4)	O35—Cu2—O53—N51	−129.0 (2)
O34—Cu2—N21—C25	179.99 (14)		

Symmetry codes: (i) −*x*, −*y*+2, −*z*+1; (ii) −*x*+1, −*y*+2, −*z*.

### Hydrogen-bond geometry (Å, °)

**Table:**

*D*—H···*A*	*D*—H	H···*A*	*D*···*A*	*D*—H···*A*
N3—H3A···O42^iii^	0.86	2.00	2.853 (3)	173
N23—H23A···O52^iv^	0.86	2.07	2.927 (3)	173
O14—H14A···O61^v^	0.84 (1)	1.81 (1)	2.613 (3)	158 (3)
O14—H14B···O30	0.85 (1)	1.94 (1)	2.782 (2)	178 (3)
O15—H15A···O31	0.84 (1)	1.89 (1)	2.701 (2)	160 (3)
O15—H15B···O63^v^	0.84 (1)	1.98 (2)	2.769 (3)	155 (3)
O34—H34A···O62	0.84 (1)	1.98 (1)	2.819 (3)	174 (3)
O34—H34B···O63	0.84 (1)	1.86 (1)	2.665 (3)	160 (3)
O35—H35A···O10	0.84 (3)	2.02 (3)	2.855 (2)	174 (3)
O35—H35B···O15^vi^	0.84 (3)	2.11 (3)	2.938 (3)	166 (3)
O61—H61A···O11^vii^	0.84 (3)	1.98 (2)	2.787 (3)	161 (4)
O61—H61B···O62^vi^	0.85 (3)	2.22 (4)	2.770 (3)	123 (4)
O62—H62A···O31^vii^	0.84 (1)	1.94 (1)	2.777 (3)	173 (4)
O62—H62B···O52	0.84 (1)	2.24 (2)	3.023 (3)	154 (3)
O63—H63A···O11^vii^	0.84 (1)	1.93 (1)	2.751 (3)	167 (3)
O63—H63B···O43	0.84 (3)	2.07 (3)	2.898 (3)	170 (3)
O63—H63B···O42	0.84 (3)	2.49 (2)	3.120 (3)	133 (3)

Symmetry codes: (iii) −*x*, −*y*+1, −*z*+1; (iv) −*x*+1, −*y*+1, −*z*; (v) *x*−1, *y*, *z*; (vi) *x*+1, *y*, *z*; (vii) *x*, *y*−1, *z*.

## References

[bb1] Driessen, W. L., Rehorst, D., Reedijk, J., Mutikainen, I. & Turpeinen, U. (2005). *Inorg. Chim. Acta*, **358**, 2167–2173.

[bb2] Gasque, L., Olguín, J. & Bernès, S. (2005). *Acta Cryst.* E**61**, m274–m276.

[bb3] Gasque, L., Ugalde-Saldívar, V. M., Membrillo, I., Olguín, J., Mijangos, E., Bernès, S. & González, I. (2008). *J. Inorg. Biochem.* **102**, 1227–1235. 10.1016/j.jinorgbio.2007.12.032 18291531

[bb4] Macrae, C. F., Edgington, P. R., McCabe, P., Pidcock, E., Shields, G. P., Taylor, R., Towler, M. & van de Streek, J. (2006). *J. Appl. Cryst.* **39**, 453–457.

[bb5] Mendoza-Díaz, G., Driessen, W. L., Reedijk, J., Gorter, S., Gasque, L. & Thompson, K. R. (2002). *Inorg. Chim. Acta*, **339**, 51–59.

[bb6] Sheldrick, G. M. (2008). *Acta Cryst.* A**64**, 112–122. 10.1107/S0108767307043930 18156677

[bb7] Siemens (1996). *XSCANS* Siemens Analytical X-ray Instruments Inc., Madison, Wisconsin, USA.

[bb8] Sosa, A. M., Ugalde-Saldívar, V. M., González, I. & Gasque, L. (2005). *J. Electroanal. Chem.* **579**, 103–111.

